# Relating size and functionality in human social networks through complexity

**DOI:** 10.1073/pnas.2006875117

**Published:** 2020-07-20

**Authors:** B. J. West, G. F. Massari, G. Culbreth, R. Failla, M. Bologna, R. I. M. Dunbar, P. Grigolini

**Affiliations:** ^a^Office of the Director, Army Research Office, Research Triangle Park, NC 27709;; ^b^Center for Nonlinear Science, University of North Texas, Denton, TX 76203-1427;; ^c^Departamento de Ingeniería Eléctrica-Electrónica, Universidad de Tarapacá, 6-D Arica, Chile;; ^d^Department of Experimental Psychology, University of Oxford, OX2 6GG Oxford, United Kingdom

**Keywords:** Dunbar number, allometry relation, network calculations, complexity, functionality/size

## Abstract

Extensive empirical evidence suggests that there is a maximal number of people with whom an individual can maintain stable social relationships (the Dunbar number). We argue that this arises as a consequence of a natural phase transition in the dynamic self-organization among *N* individuals within a social system. We present the calculated size dependence of the scaling properties of complex social network models to argue that this collective behavior is an enhanced form of collective intelligence. Direct calculation establishes that the complexity of social networks as measured by their scaling behavior is nonmonotonic, peaking around 150, thereby providing a theoretical basis for the value of the Dunbar number. Thus, we establish a theory-based bridge spanning the gap between sociology and psychology.

## Significance

Dunbar hypothesized, on the basis of empirical evidence, that a typical individual can have a stable relation with at most 150 other people. We establish that this number results from the internal dynamics of a complex network. Two network models having phase transitions are used to determine the optimal size for the most efficient information exchange. Such criticality generates intermittent events, with time intervals between successive events being independent (renewal) and scaling. The scaling index depends nonmonotonically on network size and direct calculations show that the index is maximum for networks the size of the Dunbar number and provides maximal information exchange efficiency. This result provides a theory-based bridge to span the conceptual gap between psychology and sociology.

Dunbar hypothesized, on the basis of archeological, evolutionary, and neurophysiological evidence, that 150 is the limit on the number of people with whom a typical person can maintain stable social relationships (1, 2). We suggest that this is a consequence of internal dynamics producing self-organized criticality within a social network consisting of *N* people. We use two distinct complex network models of social group dynamics that lead to phase transitions (3), termed criticality in the physics literature, to determine the optimal size of networks and compare this with the Dunbar number. Such criticality generates intermittent events, with time intervals between successive events being independent (renewal) and having an inverse power law distribution. The inverse power law index in both network models is shown herein by direct calculation to increase rapidly in magnitude from 0.5, reach a maximum of ∼0.67, and then decrease slowly back to 0.5, as the size of the network increases. This nonmonotonic dependence of the scaling index on network size is a signature of complexity (4) and is used to argue that the collective social behavior at criticality supports optimal information transmission within the group. Consequently, the time to transmit information between generic complex networks is minimal when both system sizes coincide with the predicted Dunbar number. Thus, the calculations presented herein yield a theory-predicted value of the maximum group size that closely agrees with the empirical Dunbar number, as well as showing that networks of this size have optimal information transmission properties. These results provide a theory-based bridge that uses network science model calculations to span the current conceptual gap between psychology and sociology.

In order for a group, organization, or living network to maintain its functionality as its size increases, macroscopic dynamic modes must emerge to replace those that no longer support the system’s evolving purpose as driven by the increase in complexity. The network’s size and functionality increase and decrease together as determined by their separate relationships to changing complexity, but not in direct proportion to one another.

Another way to view the relation between network complexity and network size is by relating the functionality of the network of interest to its complexity. West (5) established that the more sophisticated the functionality, the greater the complexity necessary to support that function. For example, the degree of complexity necessary to sustain the functionality of a modern city is, proportionately, significantly greater than that necessary to sustain the functionality of a primitive village. Consequently, we interpret the many empirical relations, between functionality and size as being the result of an implicit relation between size and complexity, with complexity being manifest through the system’s functionality. This subtle, yet ubiquitous, driving of complexity by size and in turn functionality being driven by complexity, has long been known from the study of allometry (5): Average network functionality is typically a noninteger power of average network size, without an explicit dependence on complexity.

In the recent past it has been argued that biological systems function best when their dynamics are close to criticality (6). This hypothesis is in keeping with the more general observations of Anderson (7) regarding the disconnect between microdynamics and emergent macrodynamics in complex dynamic systems that undergo phase transitions. For example, when a liquid is boiled it becomes a gas and the corresponding volume increases discontinuously as a manifestation of criticality. This universal behavior is manifest in the scaling behavior of certain system parameters called critical exponents, on which there is now a vast literature. In the complex networks used herein the universal properties are criticality, a dependence of critical parameters on network size *N*, and fluctuations due to the finite size of the network whose intensity varies as as *N*^*−1/2*^ (3).

The social organizations of primates provide a well-studied example of biological social networks. A quarter century ago Dunbar (1, 2) related measures of the average neocortical ratio *C* to mean group size *N* of a sample of 36 primate genera and constructed a version of the social brain allometry relation (SBAR):

$$N=aC^{b},$$

where *a* and *b* are the empirical constants: *a* = 1.239 and *b* = 3.389. Here, *C* is the ratio of neocortical volume to that of the total volume of the brain minus that of the neocortex. The average neocortex ratio for humans was measured to be *C* = 4.1 (8), which when inserted into the SBAR model yields an average group size of *N* = 147.8. This value was rounded off to 150 in the subsequent literature and is now called the Dunbar number.

The social brain hypothesis (2) from which the Dunbar number results, heuristically bridges the gap between psychology and sociology, in that it relates an empirical measure of cognition to the average size of a social group consisting of individuals sharing that average cognition measure through the SBAR. Although the Dunbar number is widely known, no first principles explanation as yet exists for it. Consequently, the search for a theory to predict the Dunbar number has shifted from explaining the social brain hypothesis to establishing a rationale as to why the number turns out to be ∼150.

The back and forth exchange of information between complex networks has been predicted theoretically with the principle of complexity matching (4) and is observed experimentally in, for example, turn-taking in dyadic conversations (9), the therapeutic influence of arm-in-arm walking (10), and the influence of zealots on group behavior (3). Human social networks, in particular, are best viewed as being designed to solve coordination problems (11), and these lend themselves naturally to the format sometimes known as opinion dynamics models (12, 13). The greater the complexity of a network the more information the network contains and just as an entropy gradient provides an entropic force in a physical network, a complexity gradient provides an information force between living networks. Consequently, with the brain of the individual modeled as one complex network and the social group as a second complex network, the tools of network science are used herein to provide a theoretical explanation for the value of the Dunbar number as the value of network size that optimizes information transport.

In keeping with the criticality hypothesis, it is reasonable to implement the SBAR association of functionality and size with the emergence of complexity from criticality. This is achieved dynamically by critical dynamics generating crucial events. Complexity, as measured by functionality, is manifest in the collective behavior of nonlinear dynamic networks to model cognition, using the concept of collective intelligence (14). Long-range correlations are amplified at the onset of phase transition and are often studied by means of dynamic networks that are members of the Ising universality class (15), which provides the mathematical rationale for the complex network models used in the numerical calculations presented herein. These network models at criticality generate intermittent (16) and crucial events (3, 17), which according to Alligrini et al. (18) is a manifestation of consciousness.

## Materials and Methods

Wisdom and Goldstone (19) conducted and interpreted a sequence of experiments on problem-solving tasks using the social concepts of innovation and imitation. These same two concepts are fundamental in the network science decision-making model (DMM) used in our calculation. The DMM is based on a two-state master equation for each individual in the network (3). In the DMM an isolated individual randomly switches between two states, and the random switching mechanism is identified as innovation. An individual also imitates those with whom she or he interacts so that the imitation parameter determines the influence of others on the switching time of the individual. We demonstrate by network science model calculations that *N* cooperatively interacting units generate criticality, namely, long-range correlations enabling the system to make cooperative decisions and properly respond to environmental perturbations. The efficiency of this response requires a balance between the fluctuation intensity (proportional to *N*^−1/2^) and the inverse power law regime in the limit of *N* very large. We prove that this balance occurs in the neighborhood of *N* = 150.

We use a statistical analysis of time series generated by criticality-induced intelligence, based on a method recently proposed to detect crucial events by Culbreth et al. (20) to find the criticality point in the complex network dynamical models. This method is based on converting empirical time-series data into a diffusion process from which the probability density function (*SI Appendix*) is calculated and the entropy determined. The procedure is called diffusion entropy analysis (DEA) and is used to determine the scaling behavior of the empirical process driving the diffusion. When criticality-induced intelligence (collective intelligence) becomes active, the driven process is expected to depart from ordinary diffusion signified by having a scaling index different from *δ* = 0.5. The modified DEA (MDEA) illustrated in Culbreth et al. (20) filters out the scaling behavior of infinite stationary memory, when it exists (21), and the remaining deviation of the scaling index from *δ* = 0.5 is solely due to crucial events.

The MDEA applied to the signal generated by the criticality-induced intelligence implements the original DEA in conjunction with the method of stripes. In the method of stripes the vertical axis is divided into many bins of equal size and an event, either crucial or not, is recorded when the signal moves from a given stripe to an adjacent stripe. A random walk (RW) step is triggered by such an event and makes a step of constant length forward each time an event occurs, thereby generating a diffusion-like trajectory *X(t).* This trajectory contains information on the persistence of opinion contained in the empirical time series, which can be detected by applying the MDEA method to *X(t)* determined from the RW rate equation (*SI Appendix*).

In order to explore the generality of our results, we have selected two network models generating criticality-induced intelligence that have totally different microdynamics, but both have transitions to criticality. The first is the DMM, where *N* individuals each choose between two conflicting states, which they do under the influence of their nearest neighbors (*SI Appendix*). This model falls into the Ising universality class, thereby making it possible to compare the results obtained herein to the predictions of Chialvo (22). The second model is that of swarm intelligence (SI) proposed by Vicsek et al. (23) and is also a member of the Ising universality class. These models are appropriate for describing human social networks since human (and primate) social groups exist to enable behavioral (and informational) coordination (24). They are also appropriate for modeling the way in which an individual models their own social network in the virtual world they construct within their brain. These social and neurologic networks correlate incompletely with one another, with information from the one influencing the way the other organizes itself (we adjust our model network as formulated in our mind/brain as a function of information received about observed changes in the state of the social network).

To show that network size is optimal with regard to the transfer of information, we consider two complex networks, A and B, which interact with one another at criticality. We seek the size at which they are identical, having the same size *N*. For a time *L,* the global field *ξ*_*A*_(*t*) of network A and the field *ξ*_*B*_(*t*) of network *B* are calculated and the cross-correlation between the two time series determined.

This cross-correlation experiment is done in two ways. In the first, a small percentage (5% of units of A, randomly chosen) make their choice on the basis of the choices made by their nearest neighbors and one randomly chosen unit of network B. Network B is influenced by network A through the same interaction process. As a result of this back and forth interaction the cross-correlation time is expected to be symmetric around τ = 0. This trivial observation turns out to be the case and therefore its calculation is not shown herein.

In the second case, the interaction is restricted to one direction with network A perturbing B, but no return perturbation of B on A. As a consequence of the unidirectional nature of the information flow, we expect that the cross-correlation function ought to shift to a positive time delay as the network size increases beyond the critical size. The time delay is a consequence of the information about A transmitted by 5% of them to the B network and does not instantaneously change the behavior of all of the B units. It requires a finite time to transmit the information from the one B unit receiving the information about the motion of the A units to all of the other B units. Attanasi et al. (25) made the conjecture that the transmission of information from one peripheral individual in a network who acquires some important item of environmental information (e.g., an approaching predator) to all of the other members of the network occurs through a diffusion process. Luković et al. (26) assigned an important role in information transmission to the visible crucial events and argued that the phase state of the network requires a sufficiently large number of dynamic switches.

### Material and Data Availability.

The original sources for all data used herein have been cited in the main text, *SI Appendix*, or the cited literature. The research codes used in the calculations of the figures have been cited and are available upon request from the University of North Texas authors: G.F.M., G.C., R.F., and P.G.

## Results

**Fig. 1.**
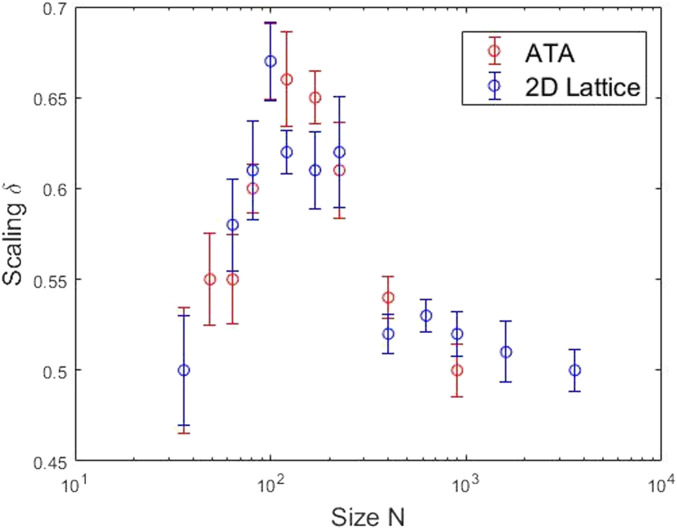
Scaling detection of the Dunbar number is obtained by calculating the nonmonotonic dependence of the scaling index *δ* on a network of size *N*. The two calculation are depicted using a DMM (3): The red circles with an ATA interaction, the blue circles with a nearest-neighbor interaction on a 2D lattice.

We evaluate first the mean field of a DMM network to produce the signal and Fig. 1 illustrates the results of that analysis. Here the calculation on the network is done in two different ways. One way is with every individual interacting with every other individual in the network (all-to-all, ATA). The other is that each individual interacts only with its nearest neighbor on a 2D lattice, with periodic boundary conditions (2D-lattice). Both calculations yield criticality at the appropriate theoretical values of the control parameter, whose critical values depend on the size of the network. Identifying the calculated value of the time rate of change of the mean field variable with the empirical time series *ξ(t)*, we generate the RW and obtain the trajectory *X(t)* to which we apply the MDEA to obtain the scaling index as a function of network size (*SI Appendix*). The resulting scaling parameter *δ* is shown in Fig. 1 and varies nonmonotonically with the size of the network. The parameter peaks, achieving a maximum value close to *δ* = 0.67, when *N* is in the vicinity of the Dunbar number 150. The scaling index falls quickly to *δ* = 0.5 to the left of the peak, for *N* < 150 and more slowly to the same value to the peak’s right, for *N* > 150.

**Fig. 2.**
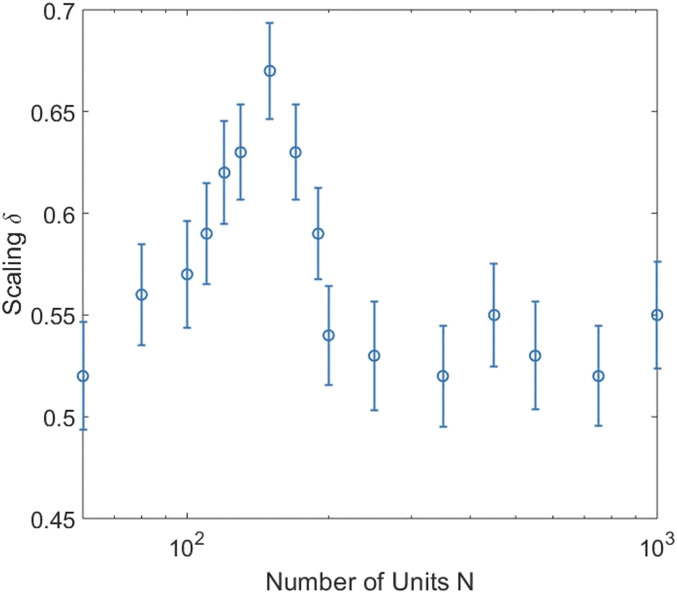
Scaling detection of the Dunbar number is obtained by calculating the nonmonotonic dependence of the scaling index δ on a network of size *N*, using the SI model of Vicsek et al. (23).

The same calculation is carried out using the SI model proposed by Vicsek et al. (23) and the results are displayed in Fig. 2. The qualitative agreement observed between the DMM in Fig. 1 and SI in Fig. 2, with respect to the nonmonotonic dependence of the scaling index on network size, is remarkable. Most noteworthy for our purposes here is that both networks display dominant peaks in the vicinity of the Dunbar number, which given the empirical value of 148.7 from the SBAR is truly astonishing.

**Fig. 3.**
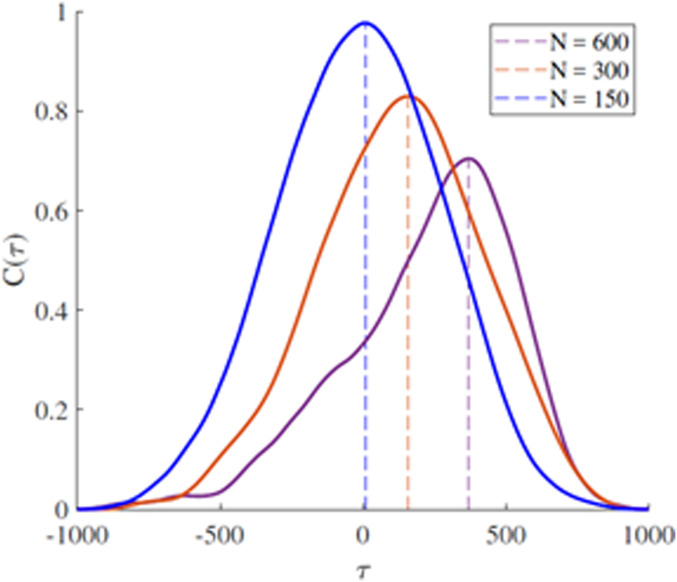
The cross-correlation function of two interacting networks, A and B, at criticality are depicted for three sizes of networks. Here 5% of A units determine their behavior by selecting the average behavior of their six nearest neighbors and one unit of B. The unit of B is influenced by the 5% of A, but otherwise interacts normally with the other members of its group. The cross-correlation function is calculated using a *z*-variable obtained by centering each time series on its time averaged value and normalizing the difference variable to the SD to obtain: $C\left(\tau \right)=\langle Z_{A}\left(t\right)Z_{B}\left(t+\tau \right)\rangle$, where the brackets denote a time average over the interval *L*.

Of course, theoretically predicting the Dunbar number does not establish that this size of the network influences the transmission of information, much less that the Dunbar size of a network optimizes the exchange of information between networks. We can, however, determine network efficiency from the cross-correlation of the time series for the perturbing network A and the perturbed network B. Fig. 3 shows that the time delay between the driven and the driving networks is extremely small when *N* = 150 (the delay time is τ ≈ 0), whereas larger networks require a finite nonzero time to reorganize and maximize their correlation. The larger the deviation in network size from the Dunbar number, the greater the delay in transmitting the information throughout the perturbed network. This is an evident signal that the Dunbar effect facilitates the transport of information from the individual who first acquires the information to all of the other individuals in the network.

As the interaction strength (*K*) between individuals in a network approaches the critical value (*K*_C_), the dynamics of individuals change from virtually independent behavior (*K* < *K*_C_) to highly organized behavior at the critical point (*K* = *K*_C_). The two-time correlation function changes from a rapid exponential relaxation of perturbations when the interaction strength is subcritical (*K* < *K*_C_), to a much slower inverse power law relaxation of perturbations at criticality (*K* = *K*_C_), and then returns to the rapid exponential relaxation when the interaction strength becomes supercritical (*K* > *K*_C_). The substantially slower relaxation perturbations at criticality entails long-range correlations, whose persistence facilitates the information transfer at criticality. The measure of this persistence is manifest in the degree to which the scaling index *δ* exceeds 0.5, but note that even at *δ* = 0.5 (the value obtained in an unbiased RW process), the network index is *µ* = 1.5, denoting that the network is still at criticality (*SI Appendix*).

## Discussion

We have established that complexity can be a hidden variable responsible for an empirical relation being directly observed between a network’s size and functionality. Recall that this was how Huxley (27) was able to “prove” the theoretical form of the allometry relation by eliminating time from two rate equations describing organs growing at different rates within the same body. Assuming that time and complexity are directly proportional (28, 29), the elimination of one is equivalent to the elimination of the other in the allometry relation between the average number of people in a social group and the average cognitive measure in the SBAR. From this we establish by numerical calculations that the Dunbar number is the optimal group size. This conclusion is here entailed by a network science theoretical model, but was reached by Dunbar using the archived evolutionary and neurophysiological data for multiple species over long time periods (1, 2).

The scaling index was used as a measure of the network’s dynamic complexity as previously done in a broad range of applications (3). Consequently, determining the dependence of the magnitude of the scaling parameter on the size of the network enabled us to ignore the microdynamics of the two models used in the calculations and determine that their complexities share the same functional dependence on network size. The remarkable result is that the two model calculations suggest that the complexity that manifests itself in the control of information transport in the dynamics of complex networks also determines the empirical SBAR and consequently the Dunbar number. In addition, the cross-correlation calculation indicates that the Dunbar number is a consequence of the information transfer being optimal for complex dynamic networks of this size. The significance of this fact in terms of the flexibility and stability of a social group cannot be overemphasized, since it provides an evolutionary advantage in which collections of weaker individuals find added strength in groups of a preferred size, whereby they can respond to a predatory attack or other ecological threats as a single collective entity and thus survive.

## Supplementary Material

Supplementary File
