# Supplementary material for: Relating size and functionality in human social networks through complexity
Source: Proc Natl Acad Sci U S A. 2020 Jul 20;117(31):18355–8. doi: 10.1073/pnas.2006875117 (PMC7414177; doi:10.1073/pnas.2006875117)
Supplement: Supplementary File [file pnas.2006875117.sapp.pdf]

SI Appendix for

Relating Size and Functionality in Human Social Networks through Complexity

B.J. West[1*], G.F. Massari[2], G. Culbreth[2], R. Failla[2], M. Bologna[3], R.I.M. Dunbar[4], and P. Grigolini[2]

1. Office of the Director, Army Research Office, Research Triangle Park, NC 27709 USA

2. Center for Nonlinear Science, University of North Texas, P.O. Box 311427, Denton, TX 76203-1427, USA

3. Departamento de Ingeniería Eléctrica-Electrónica, Universidad de Tarapacá, 6-D Arica, Chile

4. Department of Experimental Psychology, University of Oxford, Oxford OX2 6GG, UK

*Correspondence to: brucejwest213@gmail.mil

Materials and Methods

The two models described in the text, the DMM and the SI model are generators of fluctuations. In the case of DMM the fluctuations are evaluated by means of the global field, *ξ(t),* expressing the ratio of the difference between the number of units selecting ”yes” and the number of the units selecting ”no” to the total number of units. In the case of the SI model the fluctuations *ξ(t)* denote the fluctuating velocity of a swarm. It is important to stress that to detect the action of crucial events, in which the time intervals between consecutive events are renewal with inverse power law statistics and an index $\boldsymbol{\mu}$, we should convert the fluctuations *ξ(t)* into a diffusion process:

$$\boldsymbol{X(t) = \int_0^t \xi(t')dt' + X(0)}, \qquad \text{(S1)}$$

and evaluate the scaling index $\boldsymbol{\delta}$ generated by this diffusion process. If the fluctuations *ξ(t),* each fluctuation corresponding to a crucial event, are either positive or negative the scaling is given by $\boldsymbol{\delta = \frac{(\mu-1)}{2}}$ for $\mu < 2$ and by $\boldsymbol{\delta} = 0.5$ for $\mu > 2$. This is described by the blue curve Fig. S1. However, the research work done by the authors of *(SI1)* revealed that converting the negative into positive fluctuation has the effect of making the detection of the scaling index δ much more accurate. This corresponds to moving from the blue to the red curve of Fig. S1. It is important to stress that the

www.pnas.org/cgi/doi/10.1073/pnas.2006875117

results discussed in the text show that the networks at criticality move from the condition $\delta = 0.5$ generated by crucial events with inverse power law index $\mu = 1.5$ to the condition $\delta = 0.66$ where the opinion persistence gets a scaling index identical to the power index of the crucial events *(SI1)*.

In addition to this we must point out that the DMM generates fluctuations that are not genuinely crucial events but are already of diffusional nature as are the trajectories of Eq. (S1). This was made very clear by the authors of *(SI1)* showing that the proper fluctuation function to study is not $\xi(t)$, but is its time derivative instead. In the ATA case this time derivative is described by the following stochastic equation:

$$\eta = d\xi/dt = g\sinh[K(\xi+f(t))] - g\xi\cosh[K(\xi+f(t))] \quad \text{(S2)}$$

where $1/g$ defines the time scale of the single units, when they work in isolation. The random noise $f(t)$ has intensity proportional to $1/\sqrt{N}$, thereby affording information on the size of the complex network. In summary the fluctuations contained in the function $\eta(t)$ are crucial events and according to the prescriptions of *(SI1)* the diffusional trajectory $X(t)$ used for the scaling evaluation is built up by forcing the random walker to make a jump ahead of constant intensity when the fluctuation occurs. An alternative but equivalent way rests on working on the fluctuations $\xi(t)$ generated by both DMM and SI, with the help of the stripe method.

For this reason the results of the text are obtained by adopting the MDEA. This means that the fluctuation function $\xi(t)$ of DMM and SI are not directly used to define the crucial events. Using the method of the stripes we record the times at which the fluctuations $\xi(t)$ cross the border between two consecutive stripes. Should these events not be crucial, they would not contribute to the scaling emerging in the long-time limit. Furthermore, when an event occurs, the random walker always makes a step ahead of constant intensity (length). The observation of the fluctuations η in Eq. (S2) does not require the adoption of stripes but only needs converting the negative values of η into positive values.

Once the diffusional trajectory is properly created on the basis of crucial events, we use a mobile window of size l to explore the whole diffusional trajectory of length L. For any window position we record the difference between $X(t)$ at the end of the window and $X(t)$ at the beginning of the window, interpreting this as a time distance travelled by the random walker in the time

interval *l*. Due to the large number of the window positions we can define the distribution density *p(x; l)* and the Shannon entropy:

$$S(l)=-\int_{-\infty}^{\infty} \boldsymbol{p}(\boldsymbol{x},\boldsymbol{l})\boldsymbol{lnp}(\boldsymbol{x},\boldsymbol{l})\boldsymbol{d}\, x. \quad \text{(S3)}$$

Under the assumption that the above diffusion process yields the scaling structure determined by the crucial events:

$$\boldsymbol{p}(\boldsymbol{x};\boldsymbol{l}) = \frac{\mathbf{1}}{\boldsymbol{l}^{\boldsymbol{\delta}}}\boldsymbol{F}\left(\frac{\boldsymbol{x}}{\boldsymbol{l}^{\boldsymbol{\delta}}}\right), \quad \text{(S4)}$$

which when inserted into Eq.(S3) yields:

$$S(l)=A+\delta ln[l], \quad \text{(S5)}$$

where *A* is a constant determined by the function $\boldsymbol{F}\left(\frac{\boldsymbol{x}}{\boldsymbol{l}^{\boldsymbol{\delta}}}\right)$. This reasoning enables us to interpret the slope of the curve generated by graphing *S(l)* versus *ln[l]* as the scaling index δ of the diffusion process as depicted in Figure S2. It is important to notice that in the DEA the diffusion trajectory *X(t)* is realized using the experimental data *ξ(t)* directly and in the more refined version given in *(SI2)* using the method of the stripes we obtain the MDEA.

Figure S2 shows the result of this procedure in the case when MDEA is used running the stochastic equation of Eq. (S2). Similar results are obtained by using the detection of crucial events through the stripes. It is important to stress that the adoption of MDEA makes *S(l)* become a linear function of *ln[l]* in an extended time *l* region. We refer to this region as intermediate asymptotic. At short values of l the inverse power law index of the crucial events μ is not yet perceived. In the long-time *l* region the deviation from the linear behavior is usually due the small number of crucial events and consequently to statistical inaccuracy.

Bibliography for SI Appendix

SI1. B.J. West, K. Mahmoodi, P. Grigolini, *Empirical Paradox, Complexity Thinking and Generating New Kinds of Knowledge*, Cambridge Scholars Publishing, UK (2019).

SI2. G. Culbreth, B. J. West, and P. Grigolini, "Entropic Approach to the Detection of Crucial Events", *Entropy* **21**, 178-189 (2019).

Fig. S1.

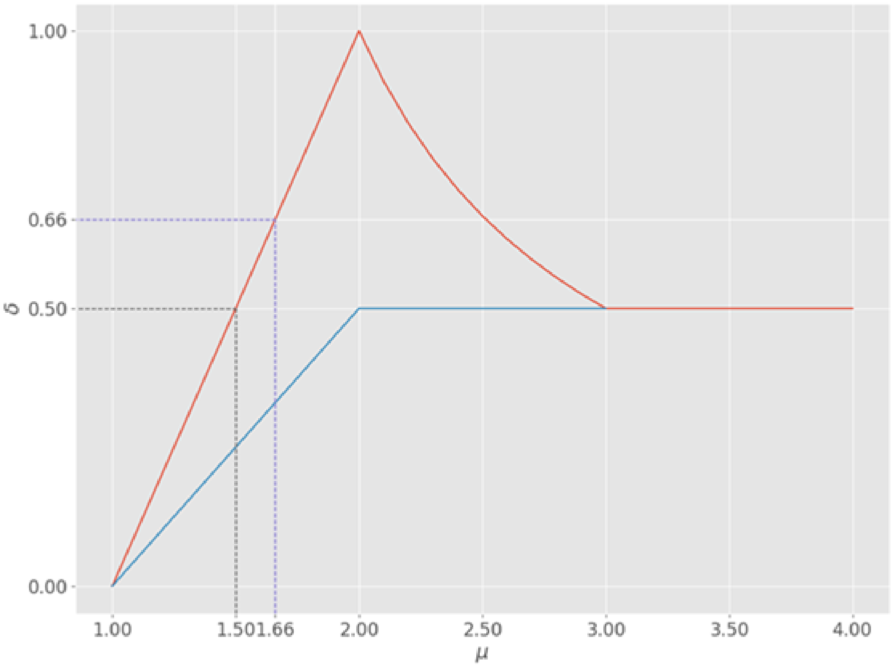

Figure S1: The relationships between diffusional scaling index δ and the crucial event index μ. The blue line corresponds to constraining the steps in DEA to always be positive. For this case: $1 < \mu < 2$, $\delta = (\mu - 1) / 2$. The red line corresponds to the case this constraint is not adopted. For that case: $1 < \mu < 2$, $\delta = \mu - 1$; $2 < \mu < 3$, $\delta = 1 / (\mu - 1)$. In this publication, we observe values of δ in the range $0.5 \leq \delta \leq 0.667$, and values μ corresponding to them.

Fig. S2.

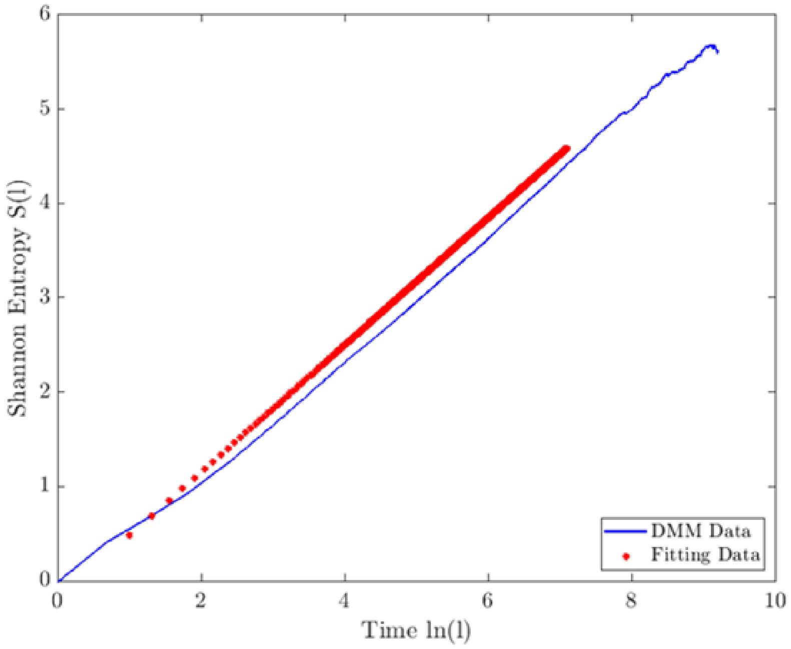

Figure S2: The time series data is generated by the DMM with ATA coupling, *N=100* and *K* very near the critical value. The result is the average over 100 trajectories, yielding an average scaling parameter of $\boldsymbol{\delta} = 0.67$ from the slopes of the MDEA curves. Note the low amplitude fluctuations around this average due to the finite size of the network.
